# Isolation, analysis and *in vitro* assessment of CYP3A4 inhibition by methylxanthines extracted from *Pu-erh* and *Bancha* tea leaves

**DOI:** 10.1038/s41598-019-50468-7

**Published:** 2019-09-26

**Authors:** Kaloyan D. Georgiev, Maya Radeva-Ilieva, Stanila Stoeva, Iliya Zhelev

**Affiliations:** 1 Department of Pharmaceutical technologies, Faculty of Pharmacy, Medical University “Prof. Dr. Paraskev Stoyanov”, Varna, 9002 Bulgaria; 2 Department of Pharmacology, toxicology and pharmacotherapy, Faculty of Pharmacy, Medical University “Prof. Dr. Paraskev Stoyanov”, Varna, 9002 Bulgaria; 3 Department of Biology, Faculty of Pharmacy, Medical University “Prof. Dr. Paraskev Stoyanov”, Varna, 9002 Bulgaria

**Keywords:** Enzyme mechanisms, Preclinical research

## Abstract

Methylxanthines, purine alkaloids found in plants, are found in beverages (coffee, tea, cocoa) and foods (chocolate and other cocoa-containing foods) commonly consumed worldwide. Members of this family include caffeine, theophylline and theobromine. Methylxanthines have a variety of pharmacological effects, and caffeine and theophylline are used as pharmaceuticals. Methylxanthines are metabolized in the liver predominantly by the enzyme CYP1A2. Their co-administration with CYP1A2 inhibitors may lead to pharmacokinetic interactions. Little is known about the possible drug interactions between caffeine and substrates of other CYP450 enzymes. In our study, methylxanthine fractions inhibited CYP3A4 in a concentration-dependent manner. Concomitant consumption of green tea with CYP3A4 substrates could increase the possibility of interactions, and this requires further clarification. The inhibition of CYP3A4 is not only due to the presence of catechin derivatives but methylxanthines may also contribute to this effect.

## Introduction

Tea is one of the most commonly consumed beverages in the world, and it has many beneficial health effects. Although they are harvested from the same plant, *Camellia sinensis*, there are many different types of tea depending on the manufacturing process, and they contain different biologically active substances. *Pu-erh* tea is a Chinese green tea produced mainly in Yunnan province. Its unique taste and aroma is achieved by the fermentation of microorganisms, such as *Aspergillus* sp., after the first drying of the leaves^1^. *Bancha* tea is one of the most commonly consumed types of green tea in Japan^2^. The process of producing *Bancha* tea is similar to that of *Sencha*, but it does not use the first two harvests, instead relying on the third and fourth flushes. Both teas contain significant amounts of methylxanthines^3^.

Methylxanthines are purine alkaloids found in plants. They are found in beverages (coffee, tea, cocoa) and foods (chocolate and other cocoa-containing foods) that are commonly consumed worldwide. The most popular and well-known methylxanthines are caffeine (1,3,7-trimethylxanthine), theophylline (1,3-dimethylxanthine) and theobromine (3,7-dimethylxanthine). Caffeine has a variety of pharmacological effects, including stimulation of the central nervous system (CNS) and cardiovascular system^4^. Theophylline is widely used in the treatment of respiratory diseases such as asthma and chronic obstructive pulmonary disease (COPD)^5^. Theobromine dilates blood vessels, especially coronary arteries, lowers blood pressure and increases heart rate. Theobromine is a more potent cardiac stimulant than caffeine^6^. Methylxanthines were tested individually as well as in fractions isolated from tea leaves. In our earlier studies, we used methylxanthine fractions and showed that they have antiproliferative activities on tumour cell lines^7^. The most likely mechanism of action of methylxanthines is antagonism at the level of adenosine receptors. Caffeine is a competitive antagonist of all adenosine receptors (A_1_, A_2A_, A_2B_ and A_3_ subtypes), but it has a high affinity for A_1_ and A_2A_ receptors, and its pharmacological effects are mainly due to the blocking of these receptor subtypes. Other proposed mechanisms, such as the mobilization of intracellular calcium and the inhibition of specific phosphodiesterases (PDEs), occur only at high, non-physiological concentrations^8^.

**Figure 1:**
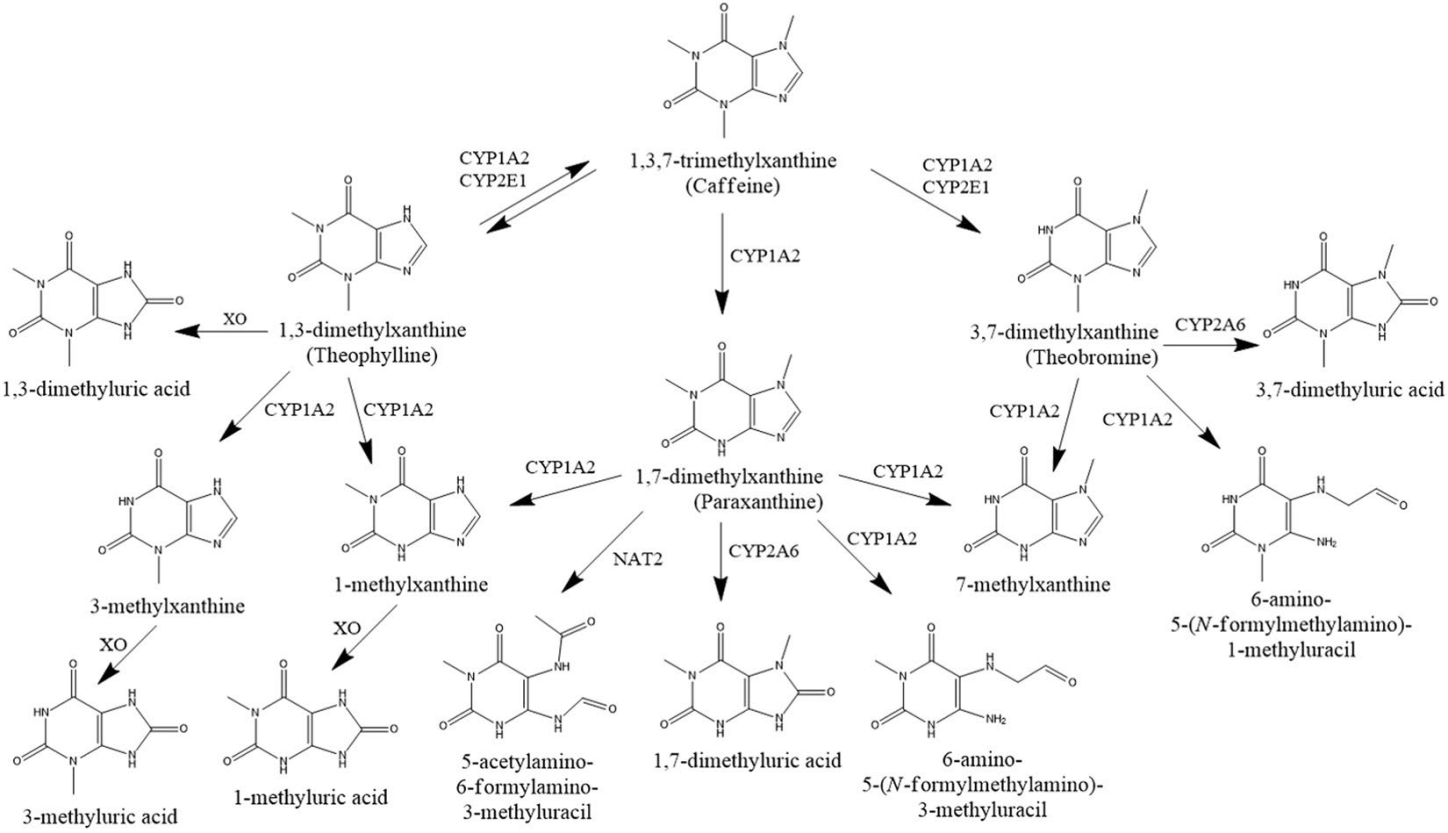
Biotransformation of methylxanthines^9^.

Methylxanthines are extensively metabolized in the liver by the cytochrome P450 (CYP450) oxidase enzyme system, mainly by demethylation, and they are excreted in human urine in the form of metabolites with less than 2% of administered caffeine being excreted unchanged^9,10^. Caffeine is metabolized predominantly by the CYP1A2 isozyme into three dimethylxanthines, paraxanthine (>80%), theobromine and theophylline^11,12^. Other enzymes involved in the biotransformation of methylxanthines are CYP2E1, CYP2A6, N-acetyltransferase 2 (NAT2) and xanthine oxidase (XO). Figure 1 shows the metabolism of caffeine and its major metabolites^9^.

Concomitant consumption of caffeine with CYP1A2 substrates (certain drugs used for cardiovascular, CNS, gastrointestinal, infectious, and other disorders) may lead to pharmacokinetic interactions at the CYP1A2 enzyme level. This may result in side effects or may hinder the treatment^13^. Little is known about the involvement of other enzymes that part in the metabolism of methylxanthines.

The aim of the present study was to analyse the methylxanthine fractions isolated from *Pu-erh* and *Bancha* tea leaves and to evaluate their potential to modulate the activity of human recombinant CYP3A4 *in vitro*.

## Results

Methylxanthine fractions were extracted using an approved standard method, as described in the Materials and Methods section, and the yields from 50 g of dried *Pu-erh* and *Bancha* tea leaves were 0.521 g (or 1.04%) and 0.475 g (or 0.95%), respectively. After isolation of the methylxanthine fractions from *Pu-erh* and *Bancha* tea leaves, HPLC-UV analysis showed that both fractions contain large amounts of caffeine (84.07% and 88.11%) and very small amounts of theobromine (0.16% and 0.11%, respectively). The *Pu-erh* tea sample contains a negligible amount of theophylline (<0.0001%), and theophylline was not detected in the *Bancha* tea sample. The results are summarized in Table 1.

**Table 1:** Determined concentrations of caffeine, theophylline and theobromine in the methylxanthine fractions isolated from *Pu-erh* and *Bancha* tea leaves and their approximate percentage.

Methylxanthine	Concentration (µg/mL) ± RSD*		Concentration (%)	
	Pu-erh	Bancha	Pu-erh	Bancha
Caffeine	525.45 ± 0.17%	220.28 ± 0.14%	~84.07	~88.11
Theophylline	0.0001 ± 0.098%	—	~<0.0001	—
Theobromine	3.88 ± 0.090%	1.06 ± 0.078%	~0.62	~0.42

For calculation of the concentrations were used six samples. *RSD – relative standard deviation.

—Below the quantification limit.

**Figure 2:**
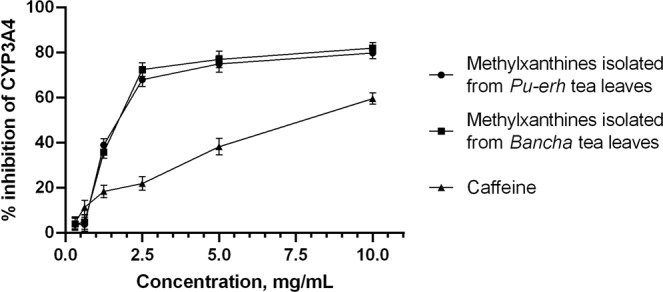
Concentration-dependent inhibition of CYP3A4 by the methylxanthine fractions. Data are presented as mean values ± S.D. of n = 6 independent experiments.

The effects of methylxanthines and caffeine alone on the activity of recombinant human CYP3A4 was analysed *in vitro* using a Vivid^®^ CYP3A4 Screening Kit. The methylxanthine fractions inhibited CYP3A4 (Fig. 2) in a concentration-dependent manner.

After analysing the results, we calculated the IC_50_ values for each fraction with a 95% confidence interval (CI). The IC_50_ value for the fraction isolated from *Pu-erh* was 1.283 mg/mL (CI: 0.9336 to 1.762 mg/mL), and that for the fraction isolated from *Bancha* was 1.351 mg/mL (CI: 1.119–1.631 mg/mL) (Table 2).

**Table 2:** Determined IC_50_ values of methylxanthine fractions isolated from *Pu-erh* and *Bancha* tea leaves and pure caffeine.

Test fractions and pure caffeine	IC50 values with 95% CI on CYP3A4
Methylxanthine fractions from *Pu-erh* tea leaves	1.28 mg/mL (CI 0.93–1.76 mg/mL)
Methylxanthine fractions from *Bancha* tea leaves	1.35 mg/mL (CI 1.11–1.63 mg/mL)
Caffeine	3,78 mg/mL (CI 1.59–8.99 mg/mL)

Values are expressed as mean with 95% CI for three independent observations.

We performed time-dependent inhibition (TDI) studies to determine the kinetic parameters. The mechanism of inhibition is probably reversible, because in the kinetic time-dependent assay, we did not observe a significant decrease in the IC_50_ values (Supplemental Figs 1 and 2).

**Figure 3:**
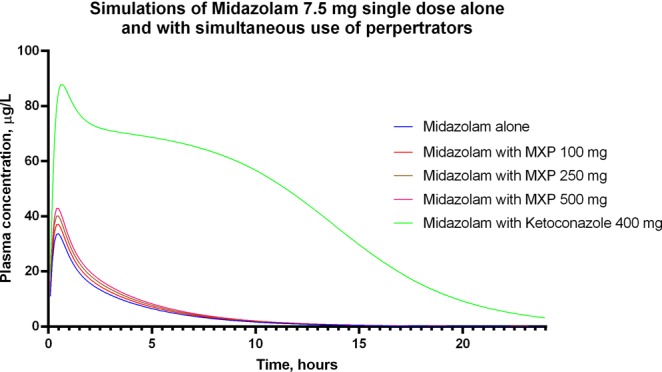
Simulation of midazolam alone (7.5 mg single dose) and the simultaneous administration of 100, 250 and 500 mg with a single dose of methylxanthine isolated from *Pu-erh*. The used positive control was ketoconazole 400 mg.

To evaluate the risk of herb-drug interactions (HDI), we performed a simulation using ADMEWORKS DDI Simulator with the methylxanthines isolated from *Pu-erh*. For the purpose of the study, we made some assumptions. First, we assumed that the mechanism of inhibition is reversible and that it is generally competitive; therefore, according to the Michaelis-Menten Kinetic equation, Ki (the inhibition constant) would be IC_50_/2, or in our case Ki = 1.28/2 = 0.64 mg/mL. Second, because it is the major component of the fraction, we used data from pure caffeine for the general information necessary for the simulation, and we only added the Ki values for inhibition of CYP3A4. For the substrate of CYP3A4, we chose midazolam because it is eliminated mainly through CYP3A4, and it is commonly used for evaluating CYP3A4 function. We simulated a single-dose administration of 7.5 mg of midazolam with concomitant consumption of 100, 250 and 500 mg of methylxanthines. For positive control we have used ketoconazole 400 mg single dose. The results are shown in Fig. 3. The simulations of the self-administration of 100, 250 and 500 mg methylxanthines are shown in Supplemental Fig. 3.

## Discussion

**Figure 4:**
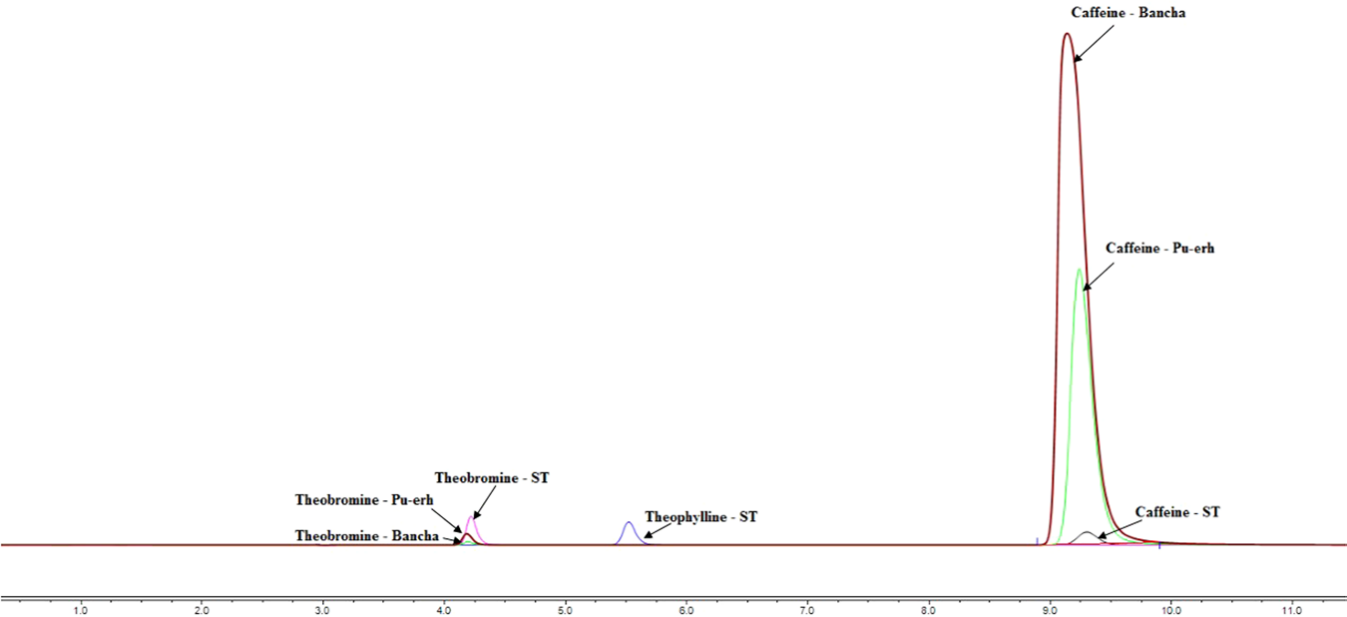
Chromatograms of caffeine, theophylline and theobromine (ST, standard substances) at concentrations of 10 µg/mL and that of the methylxanthines extracted from *Pu-erh* and *Bancha* tea leaves.

A rapid and sensitive HPLC method with UV detection was developed for the quantification of caffeine, theophylline and theobromine in *Pu-erh* and *Bancha* tea samples. Different mobile phases were used for this study^14,15^. The best separation was obtained with isocratic elution with 90% H_2_O/10% acetonitrile. Figure 4 shows the chromatograms of the reference substances (caffeine, theophylline and theobromine) and that of *Pu-erh* and *Bancha* methylxanthines.

As expected, caffeine is the main purine alkaloid in both tea fractions, accounting for 84.07% (*Pu-erh*) and 88.11% (*Bancha*), with minor differences in the percentage contents. Although in Fig. 4 the peak of caffeine at *Pu-erh* is more intense than that of *Bancha*, that is because the *Pu-erh* fraction was less dilute, 2.5 mg/mL compared to 1 mg/mL, respectively. Our study has shown results similar to those reported by Sanchez JM (2017) with regard to the contents of theobromine and theophylline^16^.

**Figure 5:**
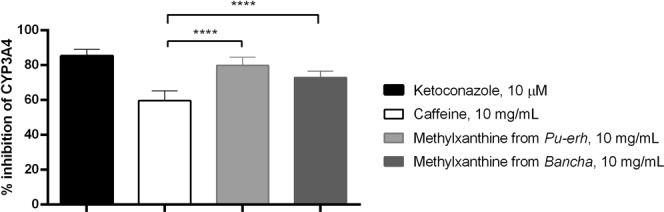
Percent inhibition of CYP3A4 by the substances and fractions at the highest concentrations evaluated in this study. Columns show mean values ± S.D. of n = 6 independent experiments. ANOVA was performed to determine the significance of the differences between groups. ****p < 0.0001.

In the second phase of our study, the potential of the methylxanthine fractions to modulate the activity of CYP3A4 isoenzyme was investigated. The biotransformation of drugs by the CYP3A4 enzyme is the major metabolic pathway for more than half of marketed drugs and therefore has the highest risk of drug interactions^13^. The methylxanthine fractions from *Pu-erh* and *Bancha* were used at a maximum concentration of 10 mg/mL and were serially diluted to a minimum concentration of 0.3125 mg/mL. Based on the HPLC chromatograms, the highest concentrations of caffeine in the *Pu-erh* and *Bancha* fractions were 8.4 mg/mL and 8.8 mg/mL, respectively. To assess the effects of the caffeine in the fractions, we used pure caffeine for comparison at a maximum concentration of 10 mg/mL and serially diluted it to a minimum concentration of 0.3125 mg/mL. For the positive control in the study, ketoconazole was used at a concentration of 10 µM, and this compound is known to produce 90% inhibition at this concentration. The results are shown in Figs 2 and 5.

As seen from the graphics, the methylxanthine fractions isolated from *Pu-erh* and *Bancha* tea leaves at concentrations of 10 mg/mL inhibit 79% and 72% of the activity of CYP3A4, respectively. Тheir effects are close to that of the positive control, ketoconazole. Their effects were significantly different than that of pure caffeine (p < 0.0001), and we can conclude that the effects of the fractions are not entirely due to the caffeine content. When reviewing articles published in the last few years, the inhibitory effects of the catechins contained in green tea are often mentioned. For example, in 2013 Misaka *et al*.^17^ reported their investigation of the activity of green tea extract (GTE) and epigallocatechin-3 gallate (EGCG) on various cytochrome enzymes, including CYP3A4, using human liver and intestinal microsomes. They established IC_50_ values of GTE for CYP3A (intestine) of 18.4 µg/mL and for CYP3A (liver) of 13.8 µg/mL, while those of EGCG were 31.1 (CYP3A, intestine) and 23.3 µM (CYP3A, liver). Satoh *et al*.^18^ studied the inhibitory potential of eight catechins from green tea on CYP3A4 and other cytochromes in human liver microsomes. The strongest effects were reported against epigallocatechin-3-gallate (EGCG) and gallocatechin-3-gallate (GCG), and the IC_50_ values were 23.7 and 40.8 µM, respectively.

In our study, the methylxanthine fractions isolated from *Pu-erh* and *Bancha* showed inhibitory activities on CYP3A4 with IC_50_ values of 1.28 mg/mL and 1.35 mg/mL. To the best of our knowledge, the possible inhibition of the activity of cytochrome CYP3A4 by methylxanthine fractions has not been reported to date. Compared to the catechins and their IC_50_ values discussed above, the effects of methylxanthines are significantly weaker. In general, we assume that the isolated methylxanthine fraction contains other components, and that the observed inhibitory effect on CYP3A4 isoenzyme is partly due to the mutual potentiation of the individual components. One study conducted by Donovan *et al*.^19^ administered decaffeinated green tea extract to healthy volunteers, and no inhibitory effects on CYP3A4 and CYP2D6 were observed. We also investigated the possibility of inhibition against other CYP isoforms by methylxanthines, such as CYP2C9 and CYP2D6. The methylxanthines from *Bancha* and *Pu-erh* showed insignificant inhibitory activities on CYP2C9 (results not shown). Research on CYP2D6 is ongoing.

The simulation of the simultaneous administration of the different doses of methylxanthine fraction from *Pu-erh* with midazolam resulted in a slight change in the time course curve, while ketoconazole used for positive control caused significant changes (Fig. 3). The calculated values of the C_max_ ratio and AUC ratio (AUCR) are presented in Table 3.

**Table 3:** C_max_ ratio and AUCR (AUC ratio) changes when midazolam (7.5 mg/single dose) is used simultaneously with different doses of Methylxanthine (100, 250 and 500 mg) and Ketoconazole (200 mg).

Different perpetrators	C_max_ ratio	AUCR
Methylxanthine 100 mg single dose	1.103	1.099
Methylxanthine 250 mg single dose	1.194	1.187
Methylxanthine 500 mg single dose	1.276	1.27
Ketoconazole 400 mg single dose	2.618	11.438

According to the EUFEPS (European Federation for Pharmaceutical Sciences) conference report, AUCR values ≥ 2 indicate high risks of drug-drug interactions (DDIs), AUCR values ≤ 1.25 are associated with low risks, and AUCR values between 1.25 and 2 indicate moderate risks of DDIs^20^. Therefore, in our case, the risk of clinically important herb-drug interactions (HDIs) is low. Although the changes observed in these simulations are insignificant, they are interesting, and some points have raised further questions. To validate the method, we have simulated the interaction between midazolam and ketoconazole. The presented results in Fig. 3 and Table 3 are supported by other publications^21,22^.

In conclusion, this *in vitro* study indicated that green tea methylxanthines might affect the activity of liver enzymes and, in particular, CYP3A4. The modulation of CYPs is well documented for green tea and its catechin fraction; nevertheless, little is known about the involvement of methylxanthines, which are also a significant factor and contribute to the biological activity of green tea. The concomitant administration of green tea and drugs metabolized predominantly by CYP3A4 could potentially alter their elimination and the safety of these drugs. The potential for interaction with methylxanthine fractions and their contribution to the observed effects on the green tea extracts require further investigation.

## Materials and Methods

### Chemicals and reagents

*Pu-erh* and *Bancha* tea leaves were purchased from the local market with quality assurance. A Vivid CYP3A4 Green Screening Kit was purchased from Antisel^®^ (Bulgaria). Caffeine, theophylline, theobromine, ketoconazole, dimethylsulfoxide (DMSO), sodium hydroxide (NaOH, >98%), sulfuric acid, chloroform (>99%), water (high-performance liquid chromatography (HPLC) grade), acetonitrile (HPLC grade, ≥99.9%) and black Costar^®^ 96-well plates were purchased from Sigma-Aldrich.

### Extraction of methylxanthines

Accurately weighed amounts of *Pu-erh* and *Bancha* tea leaves (50 g) were extracted under reflux with distilled water (250 mL) for 60 min and filtered through a Buchner funnel. The aqueous extracts were acidified with 25% sulfuric acid (5 mL) and concentrated to half of their initial volume. Then, the hot solutions were filtered and extracted four times with chloroform (50 mL) in a separating funnel. The chloroform extracts (200 mL) were washed twice with 5% sodium hydroxide solution (50 mL) and twice with distilled water (50 mL). After evaporation of the chloroform, the mixture of methylxanthines was obtained, and the percentage yield was calculated^7^.

### HPLC analysis

#### Apparatus and analytical conditions

HPLC measurements were performed with a (HPLC) Thermo Scientific UltiMate 3000 Analytical LC System equipped with a variable UV/Vis detector (Thermo Scientific Dionex UltiMate 3000 VWD-3100 Variable Wavelength Detector/VWD). HPLC separations were performed on an analytical column (Thermo Scientific AQUASIL C18, 150 mm × 4.6 mm, 5 µm), protected by an AQUASIL C18 guard column (10 mm × 4.6 mm, 5 µm) with a flow rate of 0.8 mL/min and UV detection at 274 nm. The injection volume was 20 µL. The mobile phase was a mixture of double-distilled and filtered water with acetonitrile (90:10%, v/v) in isocratic mode. The column was maintained at 30 °C. Data analysis was performed using the Thermo Scientific^®^ Chromeleon^®^ 7.2 Chromatography Data System software.

#### Standard and working solutions

Standard stock solutions of caffeine (1.0 mg/mL final concentration), theophylline (1.0 mg/mL final concentration) and theobromine (0.5 mg/mL final concentration) were separately prepared by weighing each standard substance and dissolving in hot, double-distilled water at pH 8 (adjusted with 0.1 M NaOH). Subsequently, the working solutions were prepared by serial dilutions of each standard stock solution to obtain concentrations in the range of 1.0–750.0 µg/mL in water. All stock and working standard solutions were freshly prepared before analyses.

#### Sample preparation

The *Pu-erh* tea sample was prepared by weighing 50.0 mg of the powdered methylxanthine fraction, and dissolving it in hot, double-distilled water (pH 8) to a final concentration of 2.5 mg/mL. The *Bancha* tea sample was prepared by weighing 50.0 mg of the powdered methylxanthine fraction, and dissolving it in hot, double-distilled water (pH 8) to a final concentration of 1.0 mg/mL. Before the analysis, all samples were filtered through a Sartorius RC 0.45-µm membrane filter.

#### Calibration curve

Calibration curves were constructed for each compound (caffeine, theophylline, and theobromine) using working standard solutions at eight concentrations in the range of 1.0 to 750.0 µg/mL. The linearity of the relationship between the peak area and the concentration was confirmed by the correlation coefficient (R^2^ = 0.9995). The HPLC method was validated according to the International Conference on Harmonisation (ICH) Q2(R1) Validation of Analytical Procedures: Text and Methodology^23^. The concentrations of caffeine, theophylline and theobromine were calculated from the calibration curves.

### Determination of CYP3A4 activity with Vivid P450 assay kits

The inhibitory effects of the methylxanthines isolated from *Pu-erh* and *Bancha* tea leaves and caffeine on CYP3A4 activity were determined using a Vivid^®^ CYP3A4 Green Screening Kit following the manufacturer’s instructions^24^. Stock solutions (10 mg/mL) were prepared by diluting the powdered methylxanthine fractions and caffeine (reference substance) in hot, double-distilled water. The samples in each well were mixed with a master pre-mix, containing reaction buffer, CYP450 BACULOSOMES^®^ reagent and the regeneration system, which contained glucose-6-phosphate and glucose-6-phosphate dehydrogenase. The mixture was incubated at room temperature for 20 min. Following the incubation, the CYP enzyme-specific substrate (di(benzyloxymethoxy)fluorescein (DBOMF, Vivid green substrate for CYP3A4)) and NADP^+^ were added, and the mixture was maintained at room temperature for 30 min. The reaction was stopped by the addition of 10 µM ketoconazole, and the enzyme activity was evaluated by measuring the fluorescence at excitation/emission wavelengths of 485/528 nm (BioTek Synergy 2).

### Time-dependent inhibition (TDI)

The general conditions for the conducted experiments are the same as those described above. The reaction kinetics were determined using the kinetic measurement protocol described in the manufacturer^24^ and exemplified in a study by Fairman *et al*.^25^ and this process involves fluorescence measurements taken at 5 min intervals for 30 min.

### Drug-herb interaction (DHI) simulation

ADMEWORKS DDI Simulator version 2.4. (Fudjitsu Kyushu System Limited) was used to evaluate the potential for drug-herb interactions^26^. The Simulator platform provides physicochemical and pharmacokinetic data for various CYP substrates. For the purpose of the study, we chose midazolam as the CYP3A4 substrate. The simulations were conducted using the basic PBPK (physiologically based pharmacokinetic) mechanistic model (Supplemental Fig. 4). The input parameters of MXP are shown in Table 4 and those of midazolam and ketoconazole are presented in Supplemental Tables 1 and 2.

**Table 4:** Summary of physicochemical and pharmacokinetic parameters of methylxanthines isolated form *Pu-erh* (MXP) used for DDI prediction.

Parameters	MXP	Reference/Comment
**Dosage**	100, 250 and 500 mg single dose	Assumed
**MW** – molecular weight	194.194	PubChem/Assumed to be the same as Caffeine
LogP – common logarithmic value of the octanol/water partition coefficient	−0,0403	Predicted from structure/Assumed to be the same as Caffeine
**F** – bioavailability	1	Software default value (collected from research papers)/Assumed to be the same as Caffeine
**FaFg** – fraction absorbed by the gastrointestinal tract x intestinal availability	1	Parameter calculated from other parameter information based on assumption/Assumed to be the same as Caffeine
**Fa** – fraction absorbed by the gastrointestinal tract	1	Software default value (collected from research papers)/Assumed to be the same as Caffeine
k_a_ – absorption rate constant	3.3	Parameter calculated by fitting calculation/Assumed to be the same as Caffeine
**CL**_**H**, **intr**_ – hepatic intrinsic clearance (L/h)	7.18	Parameter calculated by fitting calculation/Assumed to be the same as Caffeine
**CLr** – renal clearance (L/h)	0.233	Parameter calculated from other parameter information based on assumption/Assumed to be the same as Caffeine
**V**_**1**_ – volume of distribution in central compartment (L)	50	Parameter calculated by fitting calculation/Assumed to be the same as Caffeine
**f**_**u**,**p**_ – plasma unbound fraction	0.8	Software default value (collected from research papers)/Assumed to be the same as Caffeine
**R**_**b**_ – blood-to-plasma concentration ratio	0.8	Software default value (collected from research papers)/Assumed to be the same as Caffeine
**f**_**m**,**CYP3A4**_ - contribution ratio (f_m_ value) of CYP3A4 to the hepatic intrinsic clearance of the substrate	—	—
**Ki**,_**vitro**,**3A4 hepatic**_ – Ki,_vitro_ values of inhibitors of CYP3A4 in the liver (µg/L)	640 000	Experimental data/Measured *in vitro*

### Statistical analysis

To calculate the percentage of inhibition, we used the following equation: Percentage of inhibition = 100 − ([Signal of well (RFU, relative fluorescence units) − Blank]/[Solvent control − Blank] × 100). To build the plots and to calculate the IC_50_ values and 95% confidence intervals, we used a four-parameter logistic curve (4PL) generated in GraphPad Prism version 8.0.1 (GraphPad Software, USA), with the equation: Y = Bottom + (Top-Bottom)/(1 + 10^((LogIC50-X)*HillSlope)). The other results are expressed as percentages or means ± standard deviation (Mean ± SD) and were determined using Microsoft Excel 2013. Differences between groups were analysed using ANOVA. For every different concentration, we used six replicates, and a *P* value < 0.05 was considered statistically significant.

## Supplementary information

Dataset 1
