# Supplementary material for: Isolation, analysis and in vitro assessment of CYP3A4 inhibition by methylxanthines extracted from Pu-erh and Bancha tea leaves
Source: Sci Rep. 2019 Sep 26;9:13941. doi: 10.1038/s41598-019-50468-7 (PMC6763420; doi:10.1038/s41598-019-50468-7)
Supplement: Supplementary file 1 — Dataset 1 [file 41598_2019_50468_MOESM1_ESM.pdf]

Isolation, analysis and in vitro assessment of CYP3A4 modulation by methylxanthines extracted from Pu-erh and Bancha tea leaves

Kaloyan Georgiev[1*], Maya Radeva-Ilieva[2], Stanila Stoeva[2], Iliya Slavov[3]

[1]Department of Pharmaceutical technologies, Faculty of Pharmacy, Medical University of Varna, Varna, Bulgaria

[2]Department of Pharmacology, toxicology and pharmacotherapy, Faculty of Pharmacy, Medical University of Varna, Varna, Bulgaria

[3]Department of Biology, Faculty of Pharmacy, Medical University of Varna, Varna, Bulgaria

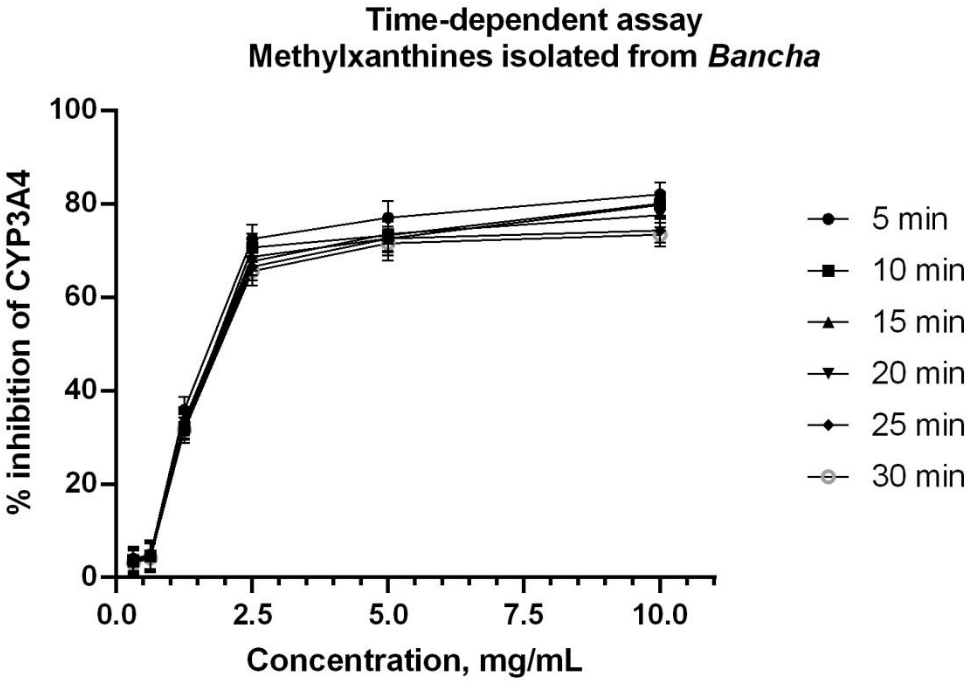

Supplemental Figure 1. Time-dependent assay of Methylxanthine isolated from *Bancha*.

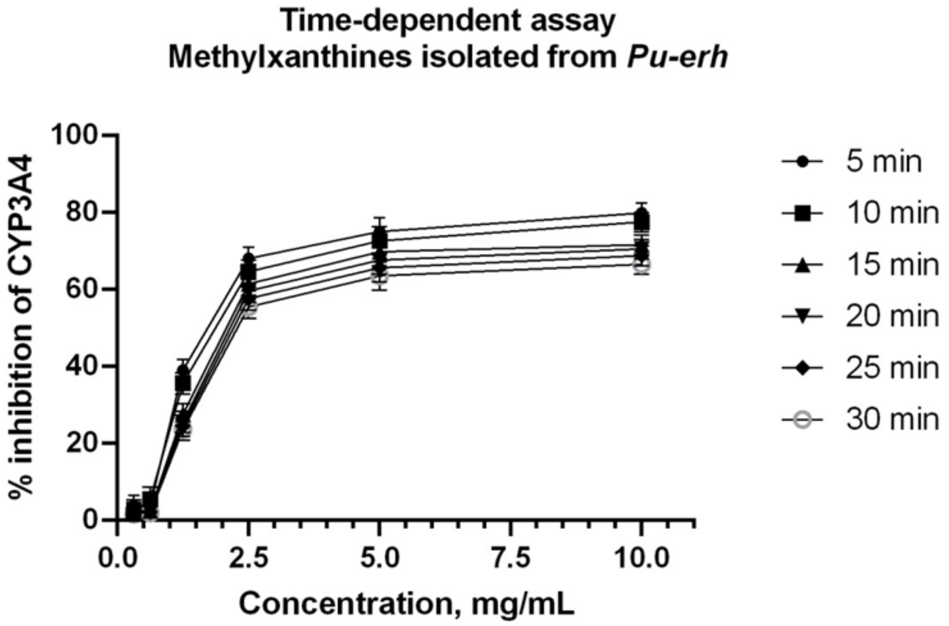

Supplemental Figure 2. Time-dependent assay of Methylxanthine isolated from *Pu-erh*.

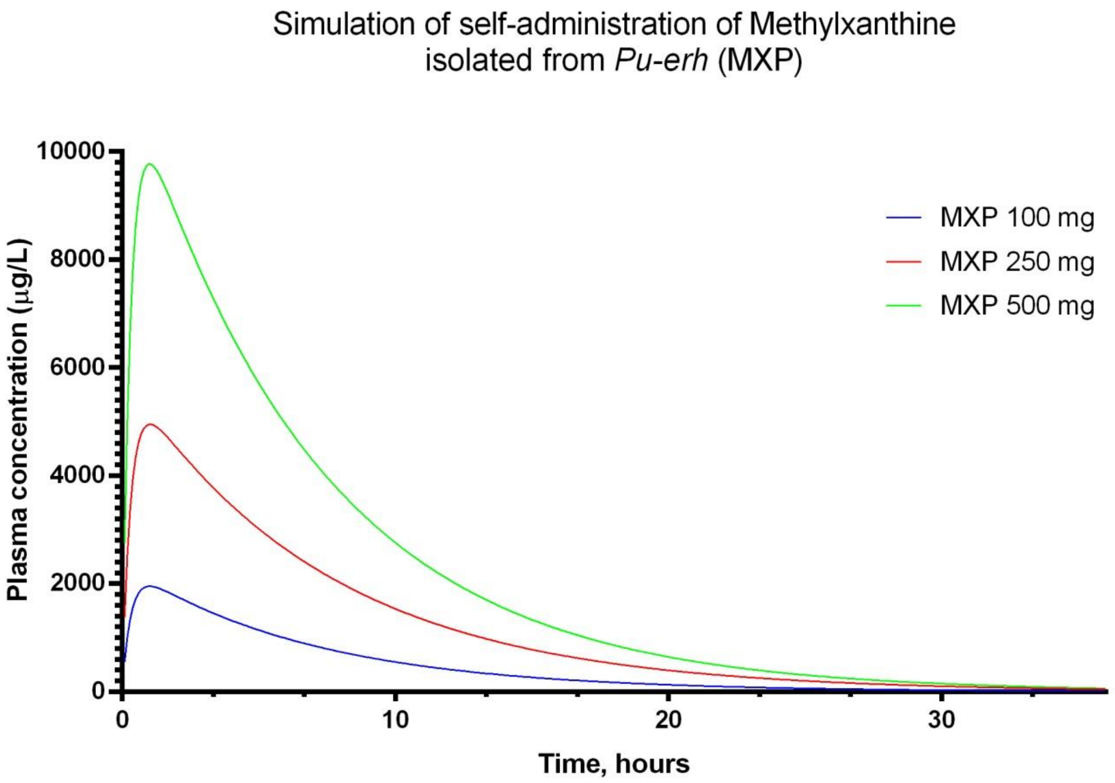

Supplemental Figure 3. Simulations of self-administration of Methylxanthine isolated from *Pu-erh*.

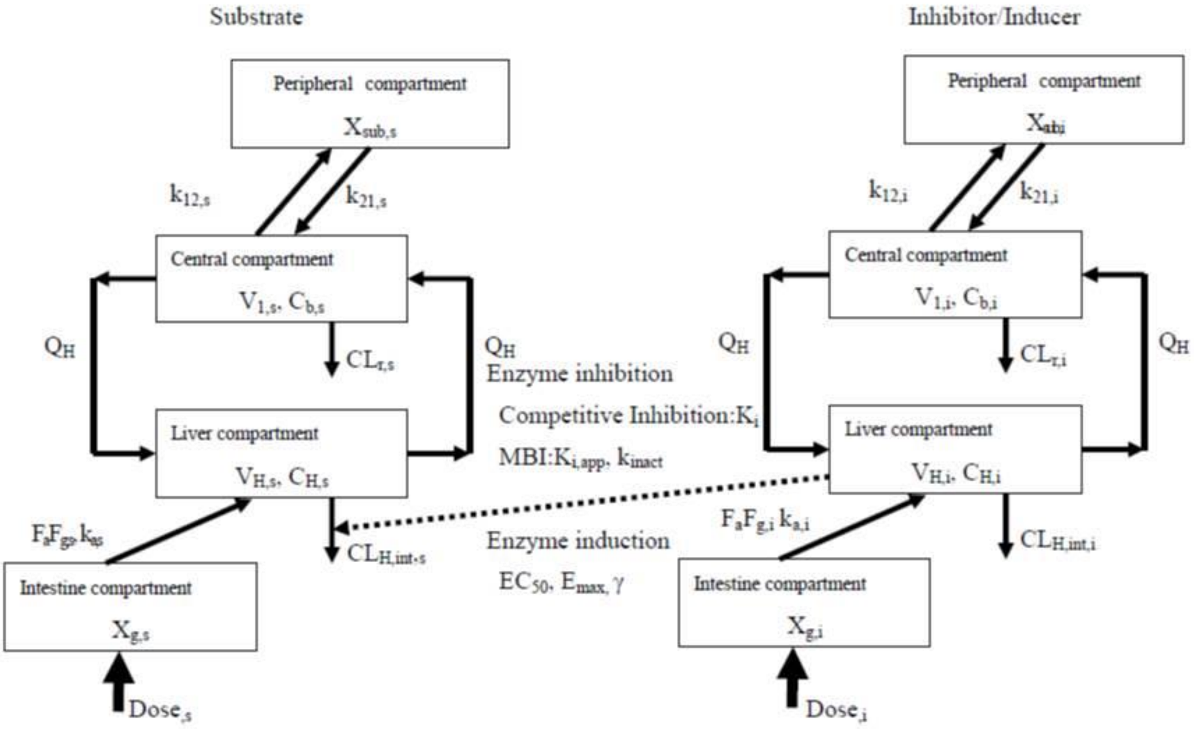

Supplemental Figure 4. Basic PBPK model used in simulations.

Parameters	*Midazolam*	*Reference*
**Dosage**	7.5 mg single dose	Assumed
**MW** – molecular weight	325.774	PubChem
**LogP** – common logarithmic value of the octanol/water partition coefficient	3.42	Predicted from structure
**F** – bioavailability	0.36	Software default value (collected from research papers)
**FaFg** – fraction absorbed by the gastrointestinal tract x intestinal availability	0.615	Parameter calculated from other parameter information based on assumption
**Fa** – fraction absorbed by the gastrointestinal tract	0.9	Software default value (collected from research papers)
$\mathbf{k_a}$ – absorption rate constant	5.66	Parameter calculated by fitting calculation
$\mathbf{CL_{H,\ intr}}$ – hepatic intrinsic clearance (L/h)	914	Parameter calculated by fitting calculation
**CLr** – renal clearance (L/h)	0.00801	Parameter calculated from other parameter information based on assumption
**Vd** – volume of distribution (L)	75.5	Parameter calculated by fitting calculation
$\mathbf{f_{u,p}}$ – plasma unbound fraction	0.05	Software default value (collected from research papers)
$\mathbf{R_b}$ – blood-to-plasma concentration ratio	0.675	Software default value (collected from research papers)
$\mathbf{f_{m,CYP3A4}}$ - contribution ratio (f_m value) of CYP3A4 to the hepatic intrinsic clearance of the substrate	0.99	Software default value (collected from research papers)

Supplemental Table 1 Summary of physicochemical and pharmacokinetic parameters of midazolam used for DDI prediction.

Parameters	*Ketoconazole*	*Reference*
**Dosage**	400 mg single dose	Assumed
**MW** – molecular weight	531.44	PubChem
**LogP** – common logarithmic value of the octanol/water partition coefficient	3.64	Predicted from structure
**F** – bioavailability	0.847	Software default value (collected from research papers)
**FaFg** – fraction absorbed by the gastrointestinal tract x intestinal availability	1	Parameter calculated from other parameter information based on assumption
**Fa** – fraction absorbed by the gastrointestinal tract	1	Software default value (collected from research papers)
$\mathbf{k_a}$ – absorption rate constant	1.08	Parameter calculated by fitting calculation
$\mathbf{CL_{H,\ intr}}$ – hepatic intrinsic clearance (L/h)	1100	Parameter calculated by fitting calculation
**CLr** – renal clearance (L/h)	0.558	Parameter calculated from other parameter information based on assumption
**Vd** – volume of distribution (L)	33	Parameter calculated by fitting calculation
$\mathbf{f_{u,p}}$ – plasma unbound fraction	0.010	Software default value (collected from research papers)
$\mathbf{R_b}$ – blood-to-plasma concentration ratio	0.632	Software default value (collected from research papers)
$\mathbf{Ki,_{vitro,3A4\ hepatic}}$ – $Ki,_{vitro}$ values of inhibitors of CYP3A4 in the liver (µg/L)	10.6	Software default value (collected from research papers)
$\mathbf{Ki,_{vivo,3A4\ hepatic}}$ – $Ki,_{vivo}$ values of inhibitors of CYP3A4 in the liver (µg/L)	0.018	Parameter calculated from other parameter information based on assumption

Supplemental Table 2 Summary of physicochemical and pharmacokinetic parameters of ketoconazole used for DDI prediction.
